# Calf circumference predicts sarcopenia and all‐cause mortality in older patients undergoing maintenance hemodialysis: A prospective cohort study

**DOI:** 10.1002/ncp.11337

**Published:** 2025-07-06

**Authors:** Yan Shen, Hongmin Qin, Xiaosu Liu, Lu Liu, Shuang Chen, Yuqi Yang, Jing Yuan, Yan Zha

**Affiliations:** ^1^ Department of Nephrology Guizhou Provincial People's Hospital Guiyang Guizhou China; ^2^ Graduate School Zunyi Medical University Zunyi Guizhou China

**Keywords:** all‐cause mortality, calf circumference, maintenance hemodialysis, older adults, sarcopenia

## Abstract

**Background:**

The role of calf circumference (CC) in predicting sarcopenia and mortality of patients undergoing maintenance hemodialysis (MHD) remains debated. This study assessed CC's predictive value, optimal threshold, and mortality association in older patients undergoing MHD.

**Methods:**

An observational cohort study was conducted on older adult patients undergoing MHD. Sarcopenia was defined by European Working Group on Sarcopenia in Older People. Logistic regression and receiver operating characteristic (ROC) analysis were used to explore the relationship between CC and sarcopenia. Kaplan‐Meier and Cox regression analyses assessed survival over 2 years.

**Results:**

A total of 979 older adult patients undergoing MHD treatment, with an average age of 73.4 years, were included in this study. The prevalence of sarcopenia was 61.1%. Male sex (odds ratio [OR], 0.17; 95% confidence interval [CI], 0.04–0.45; *P* = 0.017) and CC (OR, 0.38; 95% CI, 0.26–0.56; *P* < 0.001) were identified as independent risk factors for sarcopenia through multifactorial logistic regression analysis. ROC curves for CC and sarcopenia indicated that the optimal cutoff value for men was 32.5 cm (area under the curve [AUC], 0.904; sensitivity, 0.958; specificity, 0.841), whereas for women, it was 31.9 cm (AUC, 0.884; sensitivity, 0.922; specificity: 0.756). Kaplan‐Meier survival analysis demonstrated lower survival probabilities in patients with sarcopenia and low CC. After adjustment for multiple factors, Cox regression analysis revealed that patients in the sarcopenia group (hazard ratio [HR] = 2.411; *P* = 0.017) and those in the low‐CC group (HR = 2.045; *P* = 0.046) had significantly shorter overall survival.

**Conclusions:**

CC is an independent predictor of sarcopenia and mortality in older patients undergoing MHD.

## INTRODUCTION

Sarcopenia, an age‐related syndrome, manifests as a progressive and systemic decline in skeletal muscle mass (DSMM), strength, and function.^1^ Muscle loss is common in patients with chronic kidney disease (CKD), particularly among those undergoing maintenance hemodialysis (MHD).^2^, ^3^, ^4^ However, its ramifications extend beyond physical limitations in older adults, encompassing diminished quality of life,^5^ depression,^6^ heightened fracture risk,^6^ cardiovascular adverse events,^7^ adverse events associated with MHD, and increased rates of hospitalization and mortality.^8^ After the European Working Group on Sarcopenia in the Elderly (EWGSOP) released its initial sarcopenia consensus,^9^ numerous studies have documented the presence of sarcopenia in patients with CKD.^10^, ^11^ However, investigations specifically targeting older adults (aged ≥65 years) undergoing MHD in developing countries are lacking.

Anthropometric measurements, valued for their simplicity, noninvasiveness, and ease of acquisition, are frequently employed in clinical settings. Among them, calf circumference (CC) stands out as a prominent site for assessing SMM and, consequently, was recommended as a simplified means of evaluating muscle reduction by the Asian Working Group for Sarcopenia (AWGS) in 2019.^12^ Following this, many studies have underscored the use of CC as a valuable predictive tool for muscle atrophy in older adult patients with chronic liver disease,^13^ stroke,^14^ and fractures.^15^ Specifically, the European Sarcopenia Special Interest Group suggests using CC (<31 cm)^9^ as a key indicator for screening age‐related DSMM. However, this threshold varies because of underlying conditions, ethnicity, and environmental factors. Similarly, some studies have evaluated the effectiveness of the 2019 AWGS^12^ recommended cutoff values (<34 cm for men and <33 cm for women) in community‐dwelling older adults; however, none have assessed Asian older adult patients undergoing MHD to date.

In this cross‐sectional analysis study, we applied the EWGSOP diagnostic criteria for sarcopenia to investigate its prevalence among older adult patients undergoing MHD. Although our primary objective was to evaluate the role of CC measurements in identifying sarcopenia within this patient cohort, threshold values for CC were also proposed. Our investigations aimed to determine the relationship between CC reduction and the presence of sarcopenia in relation to all‐cause mortality.

## MATERIALS AND METHODS

### Patients

**Figure 1:**
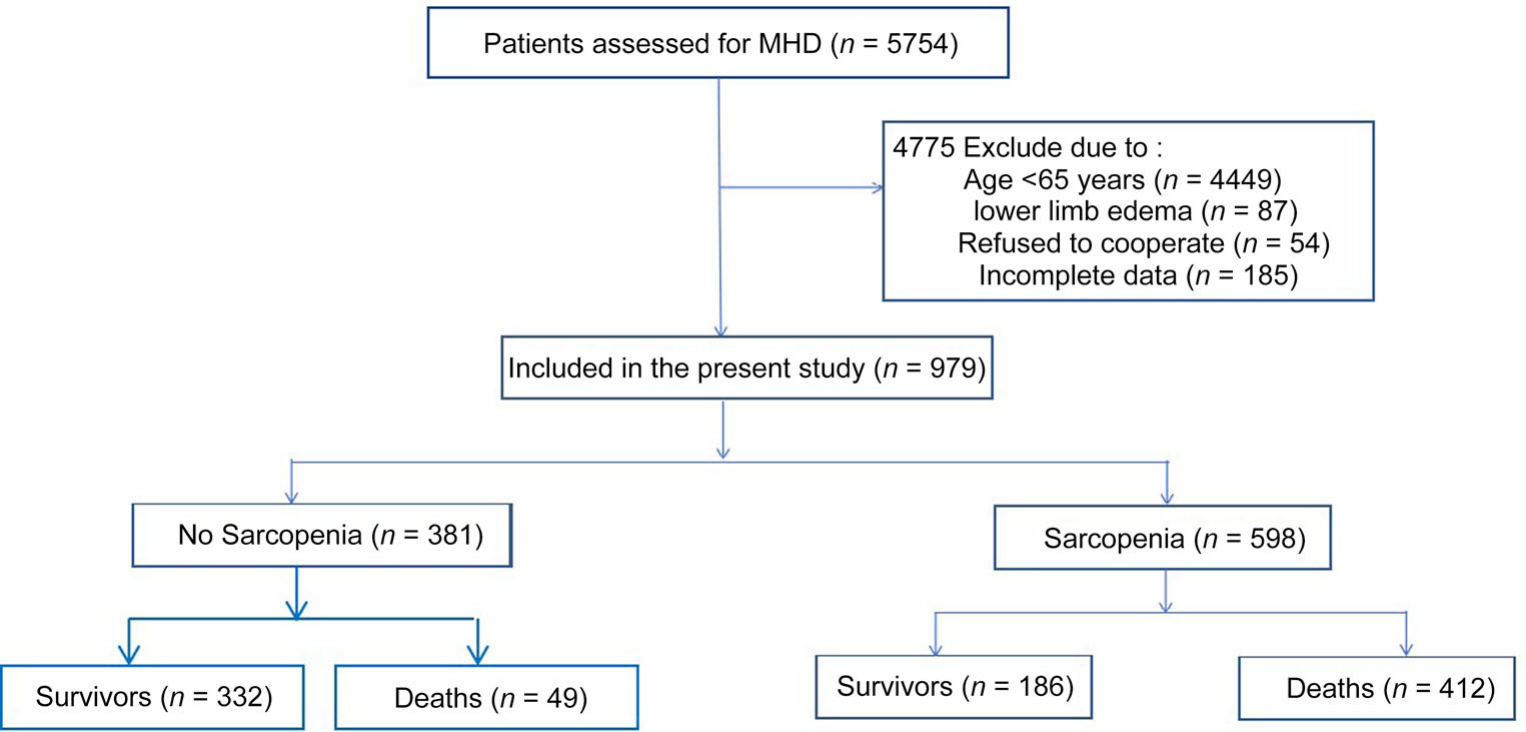
Flowchart of participant enrollment and exclusion in older adults undergoing MHD. MHD, maintenance hemodialysis.

This observational study included patients undergoing MHD across multiple tertiary hospitals in Guizhou, China, starting in 2016, and employed annual questionnaires and related tests. The data presented in this study pertain to findings from 2019. Individuals younger than 65 years, those with lower limb edema, those with missing baseline data, and those who declined to participate were excluded. The survival status of the included patients was monitored over a 2‐year period, as depicted in Figure 1. This study has been approved by the ethics committee.

### Sarcopenia diagnosis

Sarcopenia was diagnosed in accordance with the consensus of the EWGSOP^9^ and AWGS^12^ defining diagnostic criteria as low muscle strength, low muscle mass, and/or low physical performance. For muscle strength assessment, grip strength (HGS) was measured using an electronic handgrip dynamometer (EH101; Guangdong Jiamerui, China) with cutoff values <26 kg (men) and <18 kg (women). Bioelectrical impedance analysis (BIA) was performed predialysis to calculate appendicular SMM index (<7.0 kg/m^2^ for men, <5.7 kg/m^2^ for women). CC was measured postdialysis by trained staff using a nonelastic tape on the left calf's most prominent part (average of two readings). All measurements (HGS, BIA, and CC) were conducted during the same hemodialysis session. Physical performance assessment was omitted because of participants' advanced age and neurological and/or skeletal comorbidities.

### CC measurements

CC measurements were performed by trained researchers using a nonelastic measuring tape. Before dialysis initiation, patients sat with their feet flat on the floor, knees and ankles at a 90° angle, with two consecutive measurements taken on the most prominent part of the left leg; the average of the values was considered for analysis.

### Data collection

Collected variables included demographics (age, sex, smoking and alcohol history, and diabetes status), laboratory parameters (serum albumin level, hemoglobin, C‐reactive protein, and estimated glomerular filtration rate [eGFR]), anthropometrics (arm muscle circumference [AMC] and triceps skinfold thickness [TSF]), extracellular mass and total body mass, and SMM. Interrater reliability for HGS, BIA, and CC was assessed via intraclass correlation coefficients (ICCs) using a two‐way fixed‐effects model. ICC values were 0.91 (HGS), 0.89 (BIA), and 0.93 (CC), indicating excellent consistency.

All measurements (HGS, BIA, and CC) were performed by three to five trained raters during annual clinical visits (July–September). Interrater reliability was assessed via ICC using a two‐way fixed‐effects model. ICC values were 0.91 (HGS), 0.89 (BIA), and 0.93 (CC), indicating excellent consistency. Standardized training included posture alignment, anatomical landmark identification, and tool calibration.

### Statistical analysis

All data were analyzed using SPSS 25.0 statistical software. Normally distributed data are expressed as means ± standard deviation and were compared using an independent sample *t* test. Count data are presented as frequency and/or percentage and were analyzed using the chi‐square test. For the chi‐square test, if the total number of samples (*n*) was ≥40 and the theoretical number of one of the grids was between one and five (1 ≤ *T* < 5), the continuity‐corrected chi‐square test was used. If *T* was <1 or *n* was <40, Fisher exact probability method was applied. Logistic regression was employed to examine the association between sarcopenia and relevant variables.

The capacity of CC for distinguishing sarcopenia was evaluated using the area under the curve (AUC) of the receiver operating characteristic (ROC). By calculating sensitivity and specificity on the test set, an ROC curve was generated, and the AUC was calculated to assess the performance of the model. Ultimately, we selected a point on the ROC curve that balanced sensitivity and specificity, and the corresponding CC cutoff value was identified as the optimal predictor.

Survival statuses over a 2‐year period were collected, and initial comparisons between sarcopenia vs nonsarcopenia groups, as well as low‐CC vs normal‐CC groups, were conducted using Kaplan‐Meier survival analysis, highlighting differences in survival curves. Finally, Cox regression analysis was used to further explore the influence of multiple factors on overall mortality, providing a quantitative assessment of the relative hazard ratios (HRs).

### Ethical disclosures

This study has obtained approval from the local ethics committee, and all participants provided informed consent after receiving verbal and written information. The study adhered to the Declaration of Helsinki.

## RESULTS

Among the 5754 participants enrolled, 4449 individuals younger than 65 years, 87 with lower limb edema, 185 with missing baseline data, and 54 who declined to participate were excluded. Ultimately, 979 patients were enrolled in this study, with a mean age of 73.4 ± 6.2 years. Among them, 58.8% were men, and 31.3% had diabetes. Using sarcopenia diagnostic criteria, 598 (61.1%) patients were diagnosed with sarcopenia. Compared with patients without sarcopenia, those with sarcopenia were significantly older (*P* < 0.001), had lower body mass index (BMI) (*P* < 001), and had lower eGFR (*P* < 0.001). Body measurements, including CC, AMC, and TSF, were all significantly reduced in the sarcopenia group compared with the nonsarcopenia group (*P* < 0.001). Table 1 shows participant demographics, characteristics, and clinical data, as well as comparisons between the sarcopenia and nonsarcopenia groups (Table 1).

**Table 1:** The demographics, characteristics, and clinical data.

	All (N = 979)	Sarcopenia (*n* = 598)	Nonsarcopenia (*n* = 381)	Missing value	*P* value
Age, mean ± SD, years	73.4 ± 6.2	74.1 ± 6.3	72.3 ± 5.8	0	0.000
65–69, *n* (%)	395 (40.3)	217 (54.9)	178 (45.1)		
70–74, *n* (%)	237 (24.2)	138 (58.2)	99 (41.8)		
75–79, *n* (%)	187 (19.1)	132 (70.6)	55 (29.4)		
>80, *n* (%)	160 (16.3)	111 (69.4)	49 (30.6)		
Sex, *n* (%)				0	0.706
Men	576 (58.8)	330 (55.2)	246 (64.6)		
Women	403 (41.2)	268 (44.8)	135 (35.4)		
Dialysis duration, mean (IQR), month	72 (12–166)	78 (13–172)	76 (12–168)		0.842
Current smoker, *n* (%)	141 (14.4)	82 (13.7)	59 (15.5)		0.342
Habitual drinker, *n* (%)	34 (3.5)	18 (3.0)	16 (4.2)		0.275
BMI, mean ± SD, kg/m^2^	23.1 ± 3.5	22.1 ± 3.2	24.8 ± 3.2	181	<0.001*
Diabetes mellitus, *n* (%)	306 (31.3)	183 (30.6)	123 (32.3)	0	0.580
WBC, mean ± SD, 10^9^/L	6.4 ± 3.4	6.32 ± 3.80	6.64 ± 2.23	137	0.130
Hemoglobin, mean ± SD, g/L	106.1 ± 21.2	106.33 ± 21.30	105.68 ± 20.96	137	0.675
ALC, mean ± SD, 10^9^/L	1.4 ± 2.8	1.39 ± 3.27	1.27 ± 1.17	137	0.553
Serum creatinine, mean ± SD, μmol/L	688.8 ± 284.5	685.76 ± 278.8	695.32 ± 296.96	173	0.658
eGFR, mean ± SD, ml/min/1.73 m^2^	7.64 ± 3.40	7.29 ± 3.25	8.23 ± 3.56		<0.001*
Serum albumin level, mean ± SD, g/L	38.8 ± 4.8	38.6 ± 4.9	39.1 ± 4.6	173	0.194
C‐reactive protein, mean ± SD, mg/dl	12.8 ± 30.2	22.06 ± 3.20	25.5 ± 4.4	231	0.183
AMC, mean ± SD, mm	21.48 ± 2.71	20.52 ± 2.74	23.05 ± 2.66	12	<0.001*
CC, mean ± SD, mm	30.88 ± 3.31	29.27 ± 2.52	33.39 ± 2.78	0	<0.001*
TSF, mean ± SD, mm	9.26 ± 5.01	8.59 ± 4.93	10.59 ± 4.90	13	<0.001*
ECW/TBW, mean (IQR)	0.46 (0.44–0.48)	0.46 (0.44–0.49)	0.46 (0.44–0.48)	142	0.605
Handgrip strength, mean (IQR), kg	15.00 (10.50–21.20)	13.50 (9.60–18.00)	19.90 (14.08–26.88)	0	<0.001*
SMM, mean ± SD					
Men	22.61 ± 2.15	16.90 ± 2.32	23.54 ± 1.78		<0.001*
Women	14.65 ± 1.02	12.84 ± 1.65	18.15 ± 1.12		<0.001*
ASMI, mean ± SD, kg/m²				0	
Men	7.27 ± 1.13	6.52 ± 1.76	8.12 ± 1.36		<0.001*
Women	6.03 ± 1.72	5.35 ± 1.42	6.26 ± 1.50		<0.001*

*Note*: Categorical variables were compared using chi‐squared test, and continuous variables were compared using Mann‐Whitney *U* test.

Abbreviations: ALC, absolute lymphocyte count; AMC, arm muscle circumference; ASMI, appendicular skeletal muscle mass index; BMI, body mass index; CC, calf circumference; ECW/TBW, extracellular water/total body water; eGFR, estimated glomerular filtration rate; IQR, interquartile range; SMM, skeletal muscle mass; TSF, triceps skinfold thickness; WBC, white blood count.

* *P* < 0.05.

**Figure 2:**
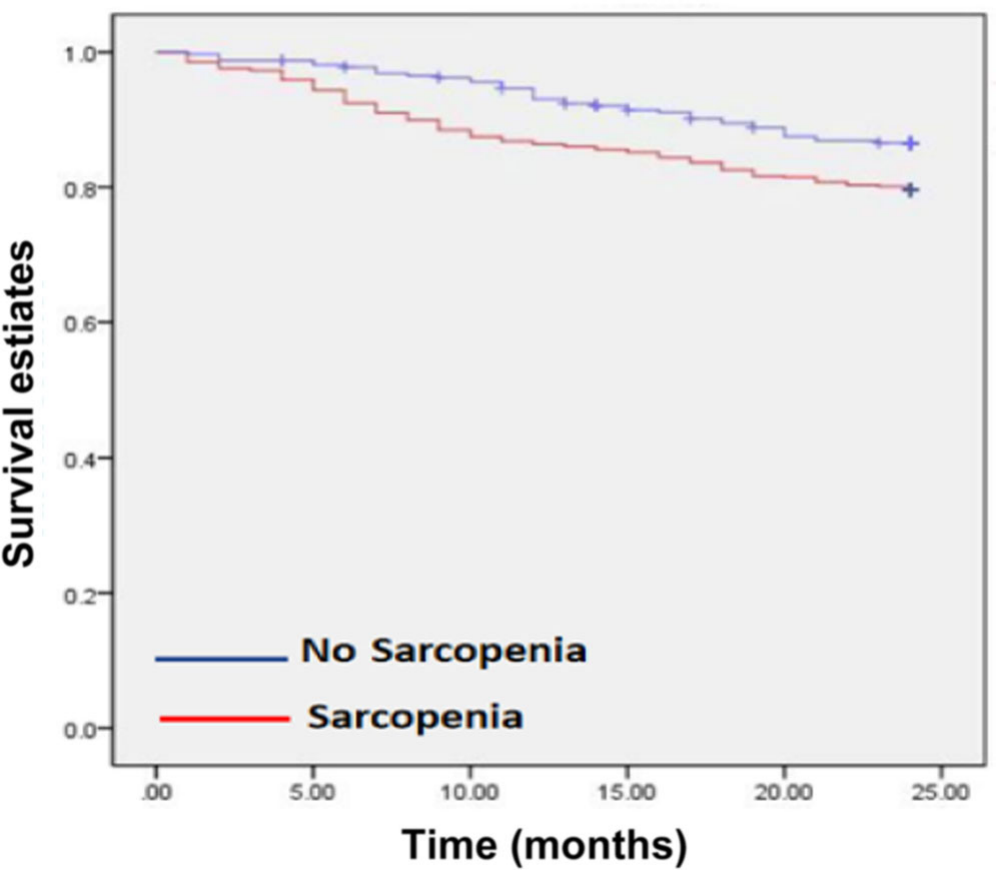
Kaplan‐Meier survival curves stratified by sarcopenia status in older patients undergoing maintenance hemodialysis (2‐year follow‐up).

During the 2‐year follow‐up period, 186 (19%) patients died, including 137 (22.3%) in the sarcopenia group and 49 (12.9%) in the nonsarcopenia group. The results of the chi‐square test indicated that the mortality rate in the sarcopenia group was significantly higher than in the nonsarcopenia group, and the difference was statistically significant (χ^2^ = 14.62; *P* = 0.0001) (Figure 2).

**Figure 3:**
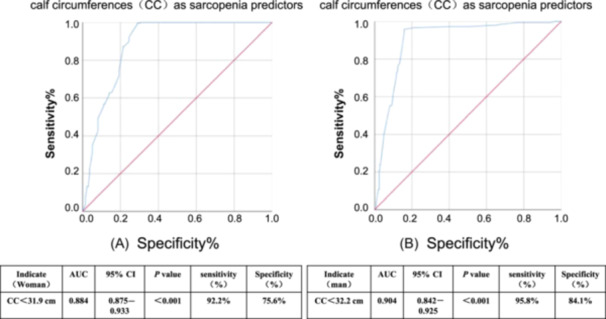
Receiver operating characteristic curve analysis of CC for predicting sarcopenia in older patients undergoing maintenance hemodialysis. (A) Men (AUC = 0.904); (B) women (AUC = 0.884). AUC, area under the curve; CC, calf circumference; CI, confidence interval.

To evaluate the risk factors associated with sarcopenia, we performed a multivariable logistic regression analysis. After adjusting for potential confounding factors, men (odds ratio [OR], 0.17; 95% confidence interval [CI], 0.04–0.45; *P* = 0.017) and a higher CC (OR, 0.38; 95% CI, 0.26–0.56; *P* < 0.001) were significantly associated with a lower risk of sarcopenia (Table 2). After establishing the significant association between higher CC and a lower risk of sarcopenia, the next step is to determine a specific CC threshold to improve clinical application and better identify high‐risk individuals. The AUC‐ROC was employed to evaluate the ability of CC to identify sarcopenia. The ROC curve indicated an optimal cutoff value of 32.5 cm for men (AUC, 0.904; sensitivity, 0.958; specificity, 0.841) and 31.9 cm for women (AUC, 0.884; sensitivity, 0.922; specificity, 0.756) (Figure 3).

**Table 2:** Multivariable logistic regression analysis for factors associated with sarcopenia.

Variable	OR (95% CI)	*P* value
Age, years	1.02 (0.92–1.13)	0.570
Men	0.16 (0.04–0.45)	0.017*
Diabetes mellitus	1.11 (0.60–2.32)	0.672
Current smoker	0.97 (0.16–5.81)	0.970
Dialysis duration, months	1.13 (0.31–13.86)	0.446
Hemoglobin, g/dl	1.01 (0.98–1.04)	0.475
eGFR, ml/min/1.73 m^2^	1.06 (0.84–1.32)	0.633
Serum albumin level, g/L	0.98 (0.88–1.09)	0.691
AMC, mm	1.01 (0.96–1.06)	0.754
ALC, mm	0.95 (0.37–2.45)	0.914
C‐reactive protein, mg/dl	0.83 (0.53–1.30)	0.413
ECW/TBW	1.34 (0.02–14.1)	0.895
BMI, kg/m^2^	1.04 (0.76–1.41)	0.893
CC, mm	0.32 (0.22–0.56)	<0.0001*

Abbreviations: ALC, absolute lymphocyte count; AMC, arm muscle circumference; BMI, body mass index; CC, calf circumference; CI, confidence interval; CRP, C‐reactive protein; ECW/TBW, extracellular water/total body water; eGFR, estimated glomerular filtration rate; OR, odds ratio.

* *P* < 0.05 indicates statistical significance.

**Figure 4:**
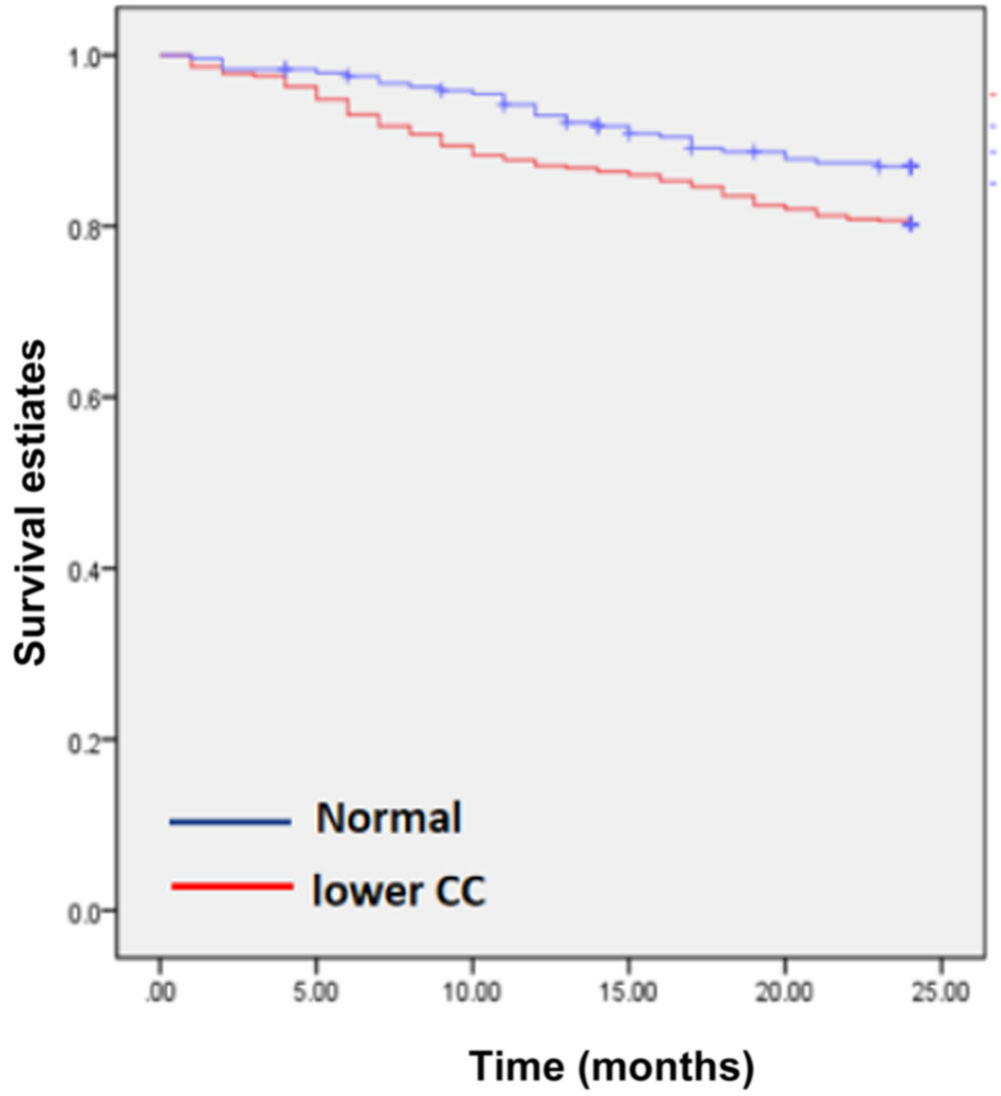
Kaplan‐Meier survival curves stratified by CC thresholds in older patients undergoing maintenance hemodialysis (2‐year follow‐up). CC, calf circumference.

To validate the role and value of the CC threshold in identifying high‐risk individuals within this population and guiding the development of intervention strategies, we used a chi‐square test to analyze the difference in mortality risk between the low‐CC group and the normal‐CC group. The results indicated that the mortality rate in the low‐CC group was lower than that in the normal‐CC group, and the difference was statistically significant (χ^2^ = 13.02; *P* = 0.0001) (Figure 4).

Survival analysis depicted a lower overall survival rate among patients with sarcopenia compared with those without sarcopenia (*P* < 0.001), as illustrated in Figure 3. Further survival analysis was conducted by categorizing patients into low‐ and normal‐CC groups based on the critical CC threshold determined earlier, showing a lower survival probability in the low‐CC group.

We conducted a multivariate regression analysis to explore the risk factors associated with mortality. First, we included sarcopenia as a variable and focused on its impact. After adjusting for potential confounders, both age and sarcopenia were found to be independently associated with overall mortality (Table 3). Next, we included low CC as a variable, and after adjusting for the same confounders, we observed that patients with a CC below the threshold had a significantly increased risk of mortality, similar to that of patients with sarcopenia (Table 4).

**Table 3:** Sarcopenia as an independent risk factor for mortality: multivariate analysis.

Mortality	HR	95% CI	*P* value
Men	0.162	0.585–1.236	0.359
Diabetes mellitus	0.075	0.622–1.385	0.714
Age	1.441	1.081–1.997	0.004*
eGFR	0.245	0.017–1.366	0.146*
Hemoglobin, g/L	0.234	0.987–1.655	0.388
Serum albumin level, g/L	0.377	0.953–1.034	0.727
ALC	0.437	0.970–1.016	0.532
CRP	1.241	0.426–2.132	0.412
ECW/TBW	0.452	0.213–1.264	0.326
BMI	0.053	0.341–2.153	0.121
Sarcopenia	1.411	1.551–2.087	0.017*

Abbreviations: ALC, absolute lymphocyte count; BMI, body mass index; CI, confidence interval; CRP, C‐reactive protein; ECW/TBW, extracellular water/total body water; eGFR, estimated glomerular filtration rate; HR, hazard ratio.

* *P* < 0.05 indicates statistical significance.

**Table 4:** Lower CC as an independent risk factor for mortality: multivariate analysis.

Mortality	HR	95% CI	*P* value
Men	0.232	0.325–2.136	0.539
Diabetes mellitus	0.015	0.322–1.578	0.632
Age	1.241	1.281–1.781	0.002*
eGFR	0.152	0.017–1.366	0.146*
Hemoglobin, g/L	0.123	0.687–1.435	0.854
Serum albumin level, g/L	0.013	0.553–1.354	0.297
ALC	0.107	0.670–1.216	0.332
CRP	1.451	0.326–2.012	0.312
ECW/TBW	0.658	0.413–1.642	0.126
BMI	0.553	0.211–2.853	0.219
Lower CC	1.782	1.051–2.756	0.007*

Abbreviations: ALC, absolute lymphocyte count; BMI, body mass index; CC, calf circumference; CI, confidence interval; CRP, C‐reactive protein; ECW/TBW, extracellular water/total body water; eGFR, estimated glomerular filtration rate; HR, hazard ratio.

* *P* < 0.05 indicates statistical significance.

## DISCUSSION

Sarcopenia is common in patients with CKD, particularly those undergoing MHD. Recent research has highlighted that the prevalence of sarcopenia among patients undergoing dialysis is significantly higher compared with that in patients not undergoing dialysis (26% vs 3%).^16^ This rate rises further in older adult patients undergoing MHD, as evidenced by epidemiological data indicating rates as high as 82.5% in Italy^17^ and 52.7% in China.^18^ In our study, sarcopenia prevalence among patients with MHD aged >65 years reached 61.1%. The primary factors contributing to this discrepancy include varying diagnostic criteria, assessment tools, fluid status anomalies in patients undergoing dialysis, and assessment timing. Furthermore, we employed BIA for SMM evaluation, whereas grip strength testing adhered to EWGSOP standards, upheld by stringent quality control measures. Because of cost limitations, we did not choose computed tomography or dual‐energy x‐ray absorptiometry (DXA) for muscle mass assessment, which makes our approach more applicable for primary healthcare settings. It is important to note that patients undergoing dialysis experience significant fluid fluctuations, especially before and after dialysis. To avoid instability in measurement results caused by the rapid reduction of fluid during dialysis, we measured CC after dialysis to more accurately reflect muscle mass, whereas we assessed other indicators (such as BIA and HGS) before dialysis to evaluate long‐term fluid burden and nutrition status, thus reflecting the most realistic condition of patients undergoing MHD. By combining predialysis and postdialysis data, we can not only minimize the interference of fluid fluctuations but also obtain a comprehensive and stable assessment of the patient's health status.

With an aging population, the increased risk of sarcopenia in elderly patients undergoing MHD significantly impacts their quality of life and prognosis. Studies have shown that individuals with sarcopenia face a mortality risk approximately one‐third higher.^10^ Because of the already high baseline mortality rate in elderly patients undergoing MHD, the risk of death increases markedly when sarcopenia is present. A 2‐year follow‐up study of patients with CKD found a mortality rate of 18% during the follow‐up period,^19^ with sarcopenia being associated with an increased risk of death (HR, 2.92; 95% CI, 1.24–6.89). Another 16‐year community‐based longitudinal prospective cohort study demonstrated a synergistic association between CKD and muscle loss, leading to an increased risk of death in patients with CKD and muscle loss. Elderly patients undergoing MHD with sarcopenia face particularly high all‐cause mortality risk.^20^ Consistent with this finding, in our study, during the 2‐year follow‐up, 19% of the patients died, and sarcopenia was associated with a significantly higher risk of death compared with patients without sarcopenia (HR, 2.411; 95% CI, 1.551–10.087). This highlights the critical importance of early prediction and identification of sarcopenia to implement preventive and intervention measures. Although BMI‐adjusted CC is commonly used to account for body composition, we chose not to employ this adjustment in our study. This decision was made because of concerns regarding the accuracy of BMI in patients with edema and the primary focus on unadjusted CC, ensuring a clearer interpretation of the relationship between CC and mortality outcomes in our cohort.

It is crucial for primary care physicians to use a simple and effective screening method to predict sarcopenia. Body measurements stand out as a fundamental approach to assessing body composition, characterized by their practicality, cost‐effectiveness, and noninvasiveness. Among these measurements, CC stands out as one of the most commonly used indicators. Additionally, the lower limbs typically have lower fat content compared with that in the upper limbs and other body areas, reducing the impact of adipose tissue when assessing muscle mass. Therefore, it holds significant value in elderly patients with strokes and chronic liver disease.^21^, ^22^ For patients undergoing MHD, in which arteriovenous fistulas are commonly established in the upper limbs, resulting in significant differences in muscle mass between the left and right upper arms, accuracy is compromised. In contrast, CC measurement offers higher precision. Previous studies have mainly focused on the correlation between CC and more complex muscle mass assessment methods, such as DXA or BIA, emphasizing the use of CC in combination with HGS and gait speed to improve the accuracy of sarcopenia screening.^23^, ^24^, ^25^, ^26^, ^27^ In our study, we found that men and a higher CC are independent protective factors against sarcopenia. Specifically, compared with women, the risk of sarcopenia in men is significantly reduced, with the likelihood of having sarcopenia being only 0.17 times that of women (an 83% reduction in risk). Contrary to studies identifying male sex as a risk factor for sarcopenia, our findings suggest male sex is protective (OR, 0.17). This discrepancy may arise from cohort‐specific factors, as follows: (1) our population included elderly patients with advanced CKD undergoing MHD, in which female patients often experience accelerated muscle loss due to hormonal changes postmenopause; (2) men in our cohort exhibited higher baseline muscle mass (CC, 33.2 ± 3.1 cm vs women's CC, 30.1 ± 2.8 cm; *P* < 0.001), potentially reflecting selection bias toward preserved muscle health in surviving men; and (3) socioeconomic factors in rural areas of Guizhou, China, such as sex‐based dietary protein intake differences, may contribute.^28^, ^29^ This suggests that physiological factors and differences in muscle mass make men less susceptible to sarcopenia under similar conditions. Additionally, higher CC significantly lowers the risk of sarcopenia, with each unit increase in CC associated with a 62% reduction in risk (OR, 0.38). The strong association between increased CC and reduced sarcopenia risk is statistically significant (*P* < 0.0001), indicating that increasing CC may help mitigate sarcopenia in patients undergoing MHD. Improving CC primarily involves enhancing lower limb muscle mass and strength through methods such as calf raises, squats, or traditional Chinese exercises like Baduanjin. However, the training program for patients undergoing MHD must consider overall health, particularly cardiovascular, bone, and fluid balance issues.

Because of racial and dietary differences, the threshold for CC may vary. For instance, a study including older adults from Turkey defined low CC as <33 cm for both men and women.^23^ In this study, we observed a statistically significant difference in CC between male and female patients undergoing MHD (*P* < 0.001), with men exhibiting higher values than women. Notably, we determined the optimal cutoff values for sarcopenia in patients undergoing MHD to be <32.5 cm for men (sensitivity, 95.8%; specificity, 84.1%) and <31.9 cm for women (sensitivity, 92.2%; specificity, 75.6%). These were very similar to the cutoffs recommended by the Global Leadership Initiative on Malnutrition criteria. However, the European Sarcopenia Task Force recommended CC <31 cm as a key screening point for DSMM in older adults. Considering the distinct dietary habits, we speculate that Chinese individuals exhibit notably lower muscle mass compared with Europeans, rendering our findings more applicable to Chinese patients undergoing MHD. In the revised AWGS guidelines, the CC cutoff values for sarcopenia are 34 cm for men and 33 cm for women, exceeding our data. This variation may stem from differences in assessment tools, study populations, and geographic regions. Our study specifically targeted patients undergoing MHD aged ≥65 years, thereby providing results that are more focused and relevant for evaluating older adult patients undergoing MHD in Guizhou, China. We found CC to be highly sensitive in predicting sarcopenia, with patients below the threshold facing a higher mortality risk similar to those with sarcopenia. This highlights the importance of CC in sarcopenia prediction. Therefore, we recommend using CC as a primary tool for predicting sarcopenia.

Several contextual factors should be interpreted with consideration. First, although BMI‐adjusted CC is commonly used to account for body composition, we deliberately avoided this adjustment because of the compromised accuracy of BMI in patients with frequent fluid overload and edema undergoing MHD.^30^ Second, participants were recruited from 13 dialysis centers in tertiary medical centers in Guiyang, Guizhou, China, which may introduce geographical biases. Because of the limitations of BIA, some patients were excluded owing to incomplete BIA test results, potentially introducing bias. Third, although predialysis BIA measurements can better reflect a patient's fluid burden, they may overestimate muscle mass. Physical performance assessments (eg, gait speed) were omitted because of participants' advanced age, neurological comorbidities, and safety concerns. Although this omission aligns with pragmatic clinical practice in frail populations, it limits direct comparison with studies using full EWGSOP criteria. Additionally, data on participants' dietary intake were not collected, which restricts our ability to analyze nutrition contributions to sarcopenia. Finally, specific causes of death were not obtained, posing challenges in interpreting mortality outcomes.

## CONCLUSIONS

In conclusion, our study provides insights into the prevalence and risk screening of sarcopenia in elderly Chinese patients undergoing MHD. Notably, CC is positively associated with sarcopenia in this population. A lower CC is linked to a higher incidence of sarcopenia and an increased risk of mortality, whereas higher CC values significantly reduce the risk of sarcopenia. This suggests that CC holds potential as a useful predictor of sarcopenia in elderly patients undergoing MHD.

## AUTHOR CONTRIBUTIONS

Yan Zha and Jing Yuan have contributed to conception and design of the research. Xiaosu Liu and Lu Liu contributed to acquisition of the data. Shuang Chen and Yuqi Yang have been involved in the interpretation of the data. Hongmin Qin and Yan Shen have read, drafted, and critically revised the manuscript. All authors agree to be fully accountable for ensuring the integrity and accuracy of the work. All authors read and approved the final manuscript.

## CONFLICT OF INTEREST STATEMENT

None declared.
